# Cardiovascular Benefits of Dietary Melatonin: A Myth or a Reality?

**DOI:** 10.3389/fphys.2018.00528

**Published:** 2018-05-17

**Authors:** Zukiswa Jiki, Sandrine Lecour, Frederic Nduhirabandi

**Affiliations:** Cardioprotection Group, Hatter Institute for Cardiovascular Research in Africa, Department of Medicine, Faculty of Health Sciences, University of Cape Town, Cape Town, South Africa

**Keywords:** antioxidant, cardiovascular diseases, hypertension, melatonin, myocardial infarction

## Abstract

The role of the diet as well as the impact of the dietary habits on human health and disease is well established. Apart from its sleep regulatory effect, the indoleamine melatonin is a well-established antioxidant molecule with multiple health benefits. Convincing evidence supports the presence of melatonin in plants and foods with the intake of such foods affecting circulating melatonin levels in humans. While numerous actions of both endogenous melatonin and melatonin supplementation are well described, little is known about the influence of the dietary melatonin intake on human health. In the present review, evidence for the cardiovascular health benefits of melatonin supplementation and dietary melatonin is discussed. Current knowledge on the biological significance as well as the underlying physiological mechanism of action of the dietary melatonin is also summarized. Whether dietary melatonin constitutes an alternative preventive treatment for cardiovascular disease is addressed.

## Introduction

The role of the diet as well as the impact of the dietary habits on human health and disease has been described since the antiquity (Skiadas and Lascaratos, 2001). In ancient Greece, excess food intake was considered as unhealthy and a cause of illness, whereas a moderate diet made of cereals, legumes, fruits, milk, honey and fish with a modest consumption of meat, confectionery and wine, as currently found in the common Mediterranean diet, was recommended as healthy (Skiadas and Lascaratos, 2001). Today, the health benefits of the Mediterranean diet are well established (Estruch et al., 2013; Afshin et al., 2014). Accordingly, supplementation of the Mediterranean diet with extra-virgin olive oil or nuts is associated with a reduction in the risk of major cardiovascular events among high-risk persons (Estruch et al., 2013). The health-promoting properties of the Mediterranean diet are attributed to its various food ingredients including, amongst others, flavonoids (Kruger et al., 2014) and, more recently, melatonin (Lecour and Lamont, 2011), therefore supporting the current growing interest in the dietary sources and bioactivities of melatonin (Peuhkuri et al., 2012; Johns et al., 2013; Sae-Teaw et al., 2013; Tan et al., 2014; Iriti and Varoni, 2015; Meng et al., 2017b).

Melatonin or N-acetyl-5-methoxytryptamine is a highly conserved indoleamine molecule found in all microorganisms (Hardeland and Fuhrberg, 1996), plants and animals (Reiter et al., 2001; Tan et al., 2012). Originally identified in the bovine pineal gland (Lerner et al., 1958), melatonin is also produced by a wide range of tissues including the retina, thymus, spleen, heart, muscle, liver, stomach, pancreas, intestine, placenta, testis, ovaries, bone marrow, skin and hair follicle, cerebral cortex, and striatum (Stefulj et al., 2001; Venegas et al., 2012; Acuna-Castroviejo et al., 2014). The content of melatonin in these tissues varies and decreases with age to a similar extent as its pineal production (Sanchez-Hidalgo et al., 2009; Scholtens et al., 2016). After biosynthesis, pineal melatonin is immediately released into the circulatory system and reaches all biological fluids including cerebrospinal fluid, bile, saliva, synovial fluid, semen, ovarian follicular fluid, amniotic fluid, breast milk and tears (Illnerova et al., 1993; Acuna-Castroviejo et al., 2014; Carracedo et al., 2017). In physiological conditions, the extra-pineal melatonin does not seem to significantly contribute to the circulating levels of melatonin (Acuna-Castroviejo et al., 2014).

Historically, the isolation and identification of melatonin in the bovine pineal gland in 1958 (Lerner et al., 1958) was motivated by the potential dermatological effects of the pineal gland extracts reported early in 1917 with the hypothesis that melatonin could play a major role in the skin lightening (McCord and Allen, 1917). Unfortunately, the skin lightening properties could not be further demonstrated (Lerner et al., 1958; McElhinney et al., 1994) and the project was then abandoned. In the early 1990s, the research conducted on melatonin received popular attention with the media to the point that its multiple actions including its potential anti-ageing activities were at some extent described as “a miracle” or “a mystery” due to a lack of scientific evidence (Reppert and Weaver, 1995). Also, the role of melatonin in the physiological regulation of seasonal and circadian rhythms (Arendt, 1998), its antioxidant properties (Tan et al., 1993) and the presence of its specific receptors (Hattori et al., 1995) were progressively established in various experimental models. Since then, numerous studies have demonstrated additional properties of melatonin (for review, see Acuna-Castroviejo et al., 2014; Reiter et al., 2016; Favero et al., 2017), making the wide range of actions of melatonin a reality and not a myth. It is now well established that endogenous melatonin induces multiple physiological responses in humans including, amongst others, synchronizing the circadian rhythms of the body, regulation of the sleep-wakefulness cycle, antioxidant capacity, modulation of the immune system and the cardiovascular system (Hardeland et al., 2011; Reiter et al., 2016).

The health benefits of melatonin as a nutritional supplement are widely accepted [EFSA Panel on Dietetic Products, Nutrition and Allergies (NDA), 2011]. Currently, melatonin is only prescribed for the regulation of sleeping patterns such as in the jet lag (Herxheimer and Petrie, 2002) and adult sleep disorders (Auld et al., 2017). However, growing evidence supports the multi-organ effects of melatonin (Opie and Lecour, 2016) with a therapeutic potential in cardiovascular pathologies (Sun et al., 2016; Pandi-Perumal et al., 2017), neurodegenerative diseases (Trotti and Karroum, 2016), reproductive diseases (Reiter et al., 2009), bone diseases (osteopenia, osteoporosis, and periodontal disease) (Maria and Witt-Enderby, 2014), various cancers (Reiter et al., 2017), skin diseases (Fischer et al., 2013) and metabolic disorders (Nduhirabandi et al., 2012; Navarro-Alarcon et al., 2014). Interestingly, melatonin is present in edible plants, meats, fruits, beverage and other food stuffs (Dubbels et al., 1995; Hattori et al., 1995; Hardeland and Pandi-Perumal, 2005; Stürtz et al., 2011; Tan et al., 2014; Herrera et al., 2018). Although the levels of melatonin in foods are much lower than those of melatonin given as a nutritional supplement, consumption of foods rich in melatonin significantly increases circulating melatonin levels in the range of the physiological concentrations (Maldonado et al., 2009; Johns et al., 2013; Sae-Teaw et al., 2013). However, little is known about the influence of the dietary melatonin intake on human health. In this review, evidence for cardiovascular health benefits of endogenous melatonin and melatonin supplementation as a pharmacological agent or from the diet is discussed.

## Evidence for cardiovascular benefits of endogenous melatonin and melatonin supplementation

A strong inverse relationship exists between endogenous melatonin levels and cardiovascular disease (Dominguez-Rodriguez et al., 2010). Epidemiological studies report that both nocturnal melatonin synthesis and circulating levels are reduced in patients with coronary heart disease (Brugger et al., 1995; Altun et al., 2002; Dominguez-Rodriguez et al., 2002), hypertension (Kozirog et al., 2011; Dominguez-Rodriguez et al., 2014), heart failure (Girotti et al., 2003; Dzida et al., 2013; Kimak et al., 2014; Dominguez-Rodriguez et al., 2016) and cardiovascular risk conditions such as diabetes (McMullan et al., 2013) and obesity (Mantele et al., 2012). Incidence for adverse cardiac events, including myocardial infarction (Dominguez-Rodriguez et al., 2002), sudden cardiac death (Muller et al., 1987) and cardiac arrhythmias (Siegel et al., 1992) increases in the early morning, when circulating melatonin levels are considerably lower (Altun et al., 2002). Similarly, low melatonin secretion levels are associated with a greater risk of incidence for myocardial infarction in women with increased body mass index (McMullan et al., 2017), supporting the crucial role of endogenous melatonin in cardiovascular pathologies.

Solid evidence supports the beneficial effects of melatonin supplementation in various cardiovascular pathologies (for review, see Paulis and Simko, 2007; Reiter et al., 2010; Sun et al., 2016). Since the cardiovascular benefits of melatonin have recently been reviewed elsewhere (Sun et al., 2016), only the evidence for the benefits of melatonin supplementation in hypertension, pulmonary hypertension and ischemic heart disease is summarized below.

### Melatonin and hypertension and other vascular pathologies

Endogenous and exogenous melatonin play an important role in hypertension and other vascular pathologies (Grossman et al., 2006; Paulis and Simko, 2007; Mozdzan et al., 2014; Simko et al., 2016). In animal studies, continuous light exposure or pinealectomy with a subsequent melatonin deficiency (Brown et al., 1991; Iigo et al., 1995) results in an increase in blood pressure (BP), a condition which is reversed by melatonin supplementation (Simko et al., 2014b). A similar finding is also reported in spontaneous hypertensive (Tain et al., 2010) and metabolic syndrome (Kantar et al., 2015) rats supplemented with melatonin, thereby confirming its therapeutic potential in hypertension.

In human studies, night time melatonin administration (2–5 mg/day for 3–4 weeks) reduces BP values of hypertensive men (Scheer et al., 2004; Grossman et al., 2006) or women (Cagnacci et al., 2005) as well as normotensive women (Cagnacci et al., 2005). In patients with metabolic syndrome, melatonin supplementation (5 mg/day, 2 h before bedtime) for 2 months reduces the systolic and diastolic BP, low-density lipoprotein cholesterol (LDL-C), thiobarbituric acid reactive substrates (TBARS, a marker of oxidative stress) and increases antioxidant defense (catalase activity) (Kozirog et al., 2011). These beneficial effects are also observed in patients with essential hypertension receiving medical treatment where melatonin (3 mg or 5 mg for 4 weeks) restores the normal circadian rhythm of BP (Mozdzan et al., 2014). However, in patients with postural tachycardia syndrome (characterized by an excessive increase in heart rate with upright posture accompanied by palpitations), melatonin does not affect systolic BP despite a modest decrease in standing tachycardia (Green et al., 2014).

**Figure 1:**
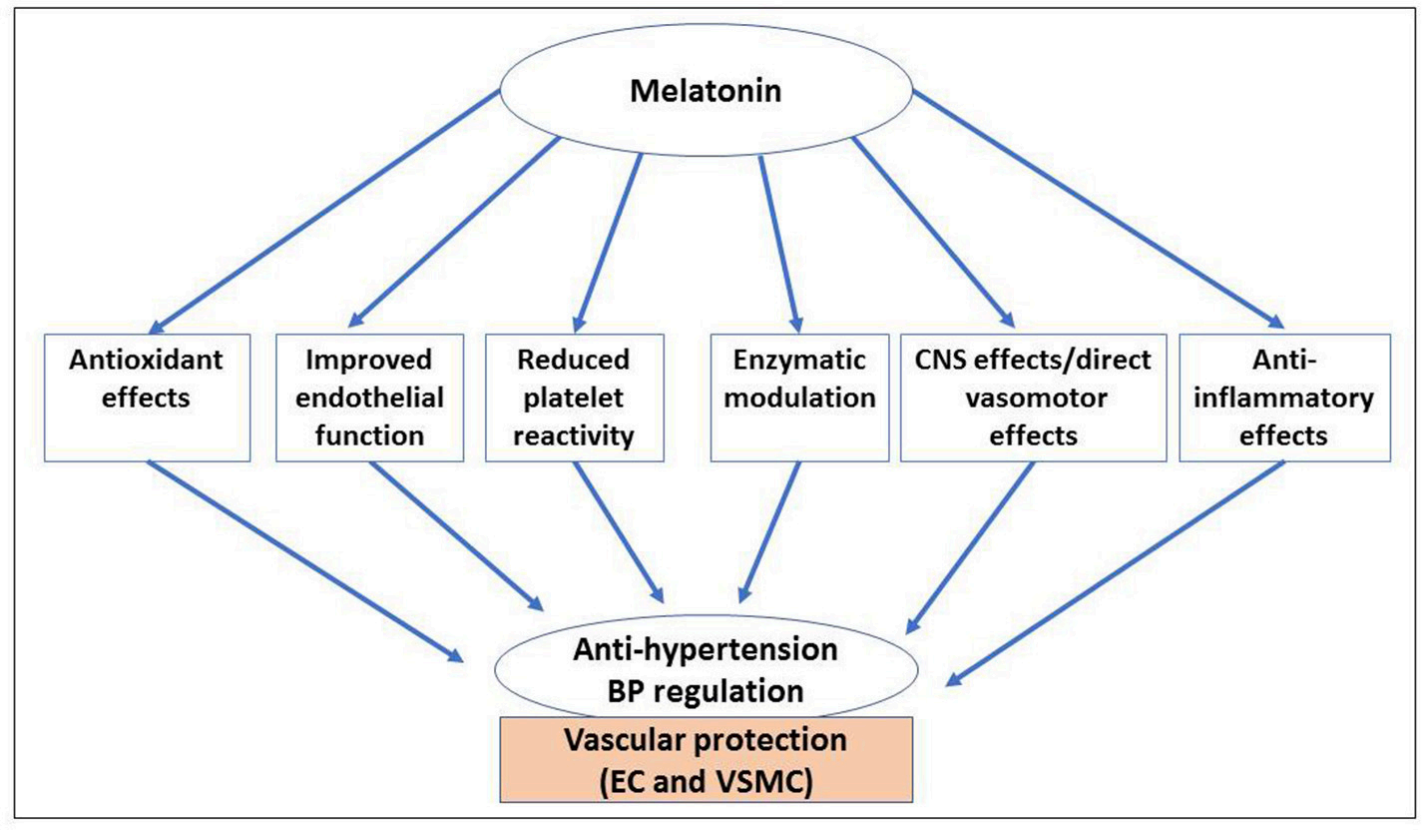
Benefits of melatonin in hypertension and vascular pathologies. Melatonin positively affects vascular function (endothelial and smooth vascular muscle cells) via its direct and indirect regulatory effects associated with its strong antioxidant, anti-inflammatory, anti-lipidemic and vasomotor properties (vasodilation), all contributing to BP regulation (anti-hypertensive effects). These effects are also associated with enzymatic modulation, improved endothelial function and a reduced platelet reactivity. BP, blood pressure; CNS, central nervous system; EC, endothelial cells; CSMC, vascular smooth muscle cells.

Figure 1 summarizes the mechanisms of actions of melatonin in hypertension and vascular pathologies. These mechanisms involve the indirect regulation of blood pressure via the central nervous system and the modulation of catecholamine secretion, the direct antioxidative and anti-inflammatory activities, the relaxation of the smooth muscle in blood vessels via α1-adrenergic receptors, nitric oxide production and calcium signaling, and the improvement of insulin signaling in insulin resistance states (Paulis and Simko, 2007; Paulis et al., 2010; Kantar et al., 2015; Agabiti-Rosei et al., 2017). In high-fat-fed rabbits, melatonin ameliorates vascular endothelial dysfunction and inflammation (Hu et al., 2013), the major contributing factors of the initiation and progression of atherosclerosis. Melatonin also improves endothelial vascular function and oxidative stress in type 2 diabetic rats (Salmanoglu et al., 2016) and insulin-resistant mice (Sartori et al., 2009). In a senescence-accelerated prone mice (SAMP8, a model of age-related vascular dysfunction), a 10-month melatonin treatment increases the expression of adiponectin and adiponectin receptor 1 in the visceral adipose tissue, the markers of vasoprotection (upregulation of eNOS and sirtuin 1 (SIRT1) and downregulation of endothelin-1 and iNOS) and inhibits aorta hypertrophy (by reducing oxidative stress and inflammation) while restoring the anticontractile effect of the perivascular adipose tissue (Agabiti-Rosei et al., 2017).

As a potential therapy in vascular disease, melatonin improves vascular function by decreasing the expression of platelets, endothelial cells adhesion molecule-1 (CD31), intercellular adhesion molecule-1 (ICAM-1), vascular cell adhesion molecule-1 (VCAM-1) and endothelin-1 (ET-1) while it increases endothelial nitric oxide synthase (eNOS), nuclear erythroid 2-related factor 2 (Nrf2), NAD(P)H quinone oxidoreductase 1 (NQO-1), catalytic glutamate cysteine ligase (GCLC) and heme oxygenase-1 (HO-1) in a rat model of a smoke-induced vascular injury (Wang et al., 2016). Similarly, in a population smoking more than 10 cigarettes per day for at least 1 year, supplementation of melatonin (3 mg/day for 2 weeks) reduces the concentration of fibrinogen and free fatty acids, the expression of ICAM-1, VCAM-1 and ET-1, and increases the expressions of Nrf2 and HO-1 (Wang et al., 2016).

### Melatonin and pulmonary hypertension

Hypoxia-induced inflammation and excessive proliferation of pulmonary artery smooth muscle cells (PASMCs) play an important role in the pathological process of pulmonary hypertension and subsequent heart failure (Jin et al., 2014). Melatonin improves hypoxia-induced pulmonary hypertension by suppressing the hypoxia-induced high expression of proliferating cell nuclear antigen (PCNA), hypoxia-inducible factor-1α (HIF-1α), and nuclear factor-κB (NF-κB) (Jin et al., 2014). These effects are associated with inhibition of proliferation of PASMCs, the levels of phosphorylation of protein kinase B (PKB/Akt) and extracellular signal-regulated kinases1/2 (ERK1/2) caused by hypoxia (Jin et al., 2014), supporting the preventive activities of melatonin via its anti-inflammatory and anti-proliferative mechanisms. Similar findings are also reported in a new born sheep model of pulmonary hypertension (Torres et al., 2015). In this model, melatonin improves the vasodilator function of small pulmonary arteries, enhancing the endothelial-and muscular-dependent pathways associated with enhanced nitric oxide-dependent and independent vasodilator components and increased bioavailability of nitric oxide in lung tissue (Torres et al., 2015).

### Melatonin and cardiac diseases

Melatonin induces multiple actions in various cardiac pathologies (Reiter et al., 2010; Lochner et al., 2013; Yang et al., 2014; Sun et al., 2016; Favero et al., 2017). In this paper, only relevant evidence in myocardial infarction, myocardial ischemia/reperfusion (I/R) injury and heart failure is included (Tables 1, 2).

**Table 1:** The effect of melatonin supplementation on cardiac diseases: animal studies.

**Animal model**		**Genre and strain**	**Age or weight**	**Sample size**	**Melatonin administration**		**Effect**	**References**
					**Dose**	**Mode and duration**		
Arrhythmias	Myocardial I/R Arrhythmias in normal rats (*in vitro*)	Male Sprague-Dawley rats	280–320 g	*n* = 10/group	1, 10, 50 μM	In the perfusate either during entire experiment or 2 min before reperfusion	Cardioprotection	Tan et al., 1998
	Myocardial I/R Arrhythmias normal rats (*ex vivo*)	Male Wistar rats	280–350 g	*n* = 6/group	1 or 10 mg/kg	I.P. at 10 min before ischemia	Cardioprotection	Lagneux et al., 2000
	Myocardial I/R Arrhythmias in pinealectomized rats (*in vivo*)	Male Wistar rats	150–200 g	*n* = 16/group	0.4 / 4 mg/kg	I.V. at 10 min before ischemia or just prior to reperfusion	Cardioprotection No effect in non-pinealectomized rats	Sahna et al., 2002
	Myocardial I/R Arrhythmias in spontaneously hypertensive and fructose-induced metabolic syndrome rats (*ex vivo*)	Male Wistar Kyoto (WKY) rats	12 wk	*n* = 12/group	50 μM	In the perfusate at reperfusion (15 min regional ischemia)	Cardioprotection	Diez et al., 2013
Myocardial I/R injury	Myocardial I/R in normal rabbits (*in vivo*)	Male New Zealand white rabbits	2.2–3.2 kg	*n* = 8/group	10 mg/kg/day	I.V. at 10 min before ischemia and 15 min before reperfusion	No effect	Dave et al., 1998
	Myocardial I/R injury in normal rats (*ex vivo*)	Male Wistar rats	250–300 g	*n* = 6/group	50 μM	In the perfusate for 15 min before ischemia and during 2h of reperfusion	Cardioprotection	Petrosillo et al., 2009
	Myocardial I/R injury in diet induced obesity rats (*ex vivo*)	Male Wistar rats	4 wk 180–220 g	*n* = 6/group	4 mg/kg/ day	Oral for 6 or 3 wk	Cardioprotection	Nduhirabandi et al., 2014
	Myocardial I/R injury in normal rats and mice (*in vivo* and *ex vivo*)	Male Wistar rats and C57BL6 mice	240–300 g (Rats) 12–16 wk (Mice)	*n* > 5/group	75 ng/L	Oral for 2 wk before I/R injury	Cardioprotection	Lamont et al., 2015
	Myocardial I/R injury in normal mice (*ex vivo*)	Male C57BL mice	12–16 wk	*n* = 6/group	75 ng/L	In the perfusate before ischemia	Cardioprotection	Nduhirabandi et al., 2016
	Myocardial I/R injury in normal rats (*ex vivo*)	Male Wistar rats	240–300 g	*n* = 36 (5–7/group)	75 ng/L	In the perfusate before ischemia	Cardioprotection	Nduhirabandi et al., 2016
	Myocardial I/R injury in a closed-chest porcine model in normal pigs (*in vivo*)	Female Danish Landrace pigs	Not given	*n* = 20	5 mg/kg (0.4 mg/mL)	IV infusion at 5 min before reperfusion for 30 min and intracoronary infusion at 1 min to reperfusion for 2 min	No effect	Ekelof et al., 2016
	Myocardial I/R injury in hyper- glycaemic rats (*in vivo*)	Male Sprague-Dawley rats	200–220 g	*n* = 6/group	10 mg/kg/day	I.V. at 5 min before and during ischemia and 4 h reperfusion	Cardioprotection	Yu et al., 2017a
	Myocardial I/R injury in diabetic rats (*in vivo*)	Male Sprague-Dawley rats	8 wk 180–220 g	*n* = 6/group	10 mg/kg/day	Oral for 5 days and I.P at 10 min before reperfusion	Cardioprotection	Yu et al., 2017b
	Myocardial I/R injury in normal fed mice (*in vivo*)	Male C57BL/6 mice	8 wk 20–22 g	*n* = 8 group	20 mg/kg/day	I.P at 10 min before reperfusion.	Cardioprotection	Zhai et al., 2017a
Heart failure	Myocardial infarction-induced heart failure in normal rats (*in vivo*)	Male Wistar albino rats	200–250 g	*n* = 6/group	10 mg/kg/day	I.P for 4 wk	Cardioprotection	Sehirli et al., 2013
	Post-infarction cardiac remodeling and dysfunction in normal mice (*in vivo*)	Male C57BL Mice	8–12 wk	*n* = 6/group	20 mg/kg/day	Oral for 1 wk before MI	Cardioprotection	Hu et al., 2017
	Isoproterenol-induced myocardial infarction in normal rats (*in vivo*)	Male Sprague-Dawley rats	10 wk 175–225 g	*n* = 6/group	10 mg/kg/day	I.P. for 1 wk	Cardioprotection	Patel et al., 2010
	Isoproterenol-induced heart failure in normal rats (*in vivo*)	Male Wistar rats	12 wk	*n* = 12/group	10 mg/kg/day	Oral for 2 or 4 wk	Cardioprotection But no effect on LV or RV hypertrophy	Simko et al., 2014a
	Pathological cardiac hypertrophy induced by transverse aortic constriction in normal mice (*in vivo*)	Male C57BL/6 mice	20–25g 8–10 wk	*n* = 85 (*n* = 10–40/group)	20 mg/kg/day	Oral for 4 or 8 wk	Cardioprotection	Zhai et al., 2017b
Pulmonary hypertension	Chronic hypoxia-induced RV hypertrophy and pulmonary hypertension (*in vivo*)	Male Sprague–Dawley rats	200–250g	*n* = 7/group	15 mg/kg/day	I.P. morning /wk prior to hypoxic and 4 wk hypoxia	Cardioprotection	Jin et al., 2014
	Monocrotaline-induced pulmonary hypertensive rats (*ex vivo* heart perfusion)	Male Long Evans rats	150–175g	*n* = 6/group	75 ng/L 6 mg/kg/day	Oral for 2 or 4 wk	Cardioprotection	Maarman et al., 2015
Hypertensive heart disease	Continuous light-induced hypertensive rats (6 wk) (*in vivo*)	Male Wistar rats	12 wk	*n* = 10/group	10 mg/kg/day	Oral for 6 wk	Cardioprotection But no effect on LV hypertrophy	Simko et al., 2014b

*I/R, ischemia reperfusion; MI, myocardial infarction; LV, left ventricular; RV, right ventricular; IV, intravenous injection; IP, intraperitoneal injection; wk, week*.

**Table 2:** The effect of melatonin supplementation on cardiac diseases: human studies.

**Type of the study**	**Sample size (*n*)**	**Age of patients (years)**	**Male/female ratio**	**Melatonin administration (dose, delivery mode and duration)**	**Effect**	**References**
A randomized triple-blinded, placebo-controlled study including patients undergoing coronary artery bypass grafting (CABG) surgery	58	58.1 ± 9.8 (42–75)	14/1	10 mg tablet once daily for 4 wk before surgery	Cardioprotection	Haghjooy-Javanmard et al., 2013
A prospective, randomized, double-blinded, placebo-controlled clinical trial including patients undergoing surgery for abdominal aortic aneurisms (AAA)	50	67 (45–80)	23/3	50 mg infusion over a 2-h period intra-operative, and oral 10 mg for the first 3 nights after surgery	Cardioprotection	Gogenur et al., 2014
A double blinded placebo-control study including patients undergoing coronary artery bypass grafting (CABG) surgery	45	52.3 (45–65) and 53.9 (45–64)	13/2 (10 mg) and 11/4 (20 mg)	10 and 20 mg, capsules once daily for 5 days before surgery	Cardioprotection	Dwaich et al., 2016
A prospective, multicenter, randomized, double-blind, placebo-controlled study for the Melatonin adjunct in the acute myocardial infarction treated with angioplasty (MARIA) trial	125	57.3 ± 10	50/13	IV: 51.7 μmol for 60 min starting immediately before percutaneous coronary intervention and IC bolus of 8.6 μmol through-PCI guiding catheter within the first 60 seconds of reperfusion	No effect	Dominguez-Rodriguez et al., 2017b
A *post-hoc* analysis of the randomized, double-blinded, placebo-controlled study for the Melatonin adjunct in the acute myocardial infarction treated with angioplasty (patients with ST-elevation myocardial infarction) (MARIA) trial	125 1st, 2nd,3rd tertiles: 41, 43, 41	1st: 54 ± 10 2nd: 58 ± 10 3rd: 60 ± 11	1st: 18/3 2nd: 20/6 3rd: 18/5	IV 51.7 μmol for 60 min starting immediately before percutaneous coronary intervention and IC bolus of 8.6 μmol through PCI-guiding catheter within the first 60 seconds of reperfusion	Cardioprotection in the 1st tertile (early after symptom onset) No effect in 2nd and 3rd tertiles	Dominguez-Rodriguez et al., 2017a
A randomized, double-blinded, placebo-controlled trial for intracoronary and systemic melatonin to patients with ST-elevation myocardial infarction (IMPACT) trial	48	61.7 (56.2–66.9)	20/3	50 mg; IC and IV infusion starting immediately after PCI with a flow rate fixed at 80 ml/h for 6 h	No effect	Ekeloef et al., 2017

*PCI, percutaneous coronary intervention; IV, intravenous; IC, Intracoronary; wk, week*.

Melatonin supplementation protects the heart in several experimental models of myocardial infarction and myocardial I/R injuries (Lochner et al., 2013; Yang et al., 2014). Tan and co-workers were first to demonstrate the beneficial effects of melatonin in isolated rat hearts subjected to cardiac I/R-induced arrhythmias: infusion of melatonin (during the period of ischemia and reperfusion or reperfusion only) reduces premature ventricular contraction and ventricular fibrillation due to occlusion and reopening of the anterior descending coronary artery (Tan et al., 1998). This finding is also reported in other animal models of cardiac arrhythmias (Lagneux et al., 2000; Sahna et al., 2002; Diez et al., 2013), confirming the therapeutic potential for melatonin in reducing the incidence of sudden cardiac death (Sahna et al., 2002). Additional studies show that short-and long-term melatonin supplementation is cardioprotective in various *in vivo* and *ex vivo* myocardial models of I/R injury (Lagneux et al., 2000; Lochner et al., 2006; Lamont et al., 2011; Nduhirabandi et al., 2011, 2014, 2016; Maarman et al., 2015; Zhai et al., 2017a,b). In these studies, administration of melatonin before or after ischemia preserves the microstructure of the cardiomyocyte and reduced myocardial I/R injury as indicated by a reduction in myocardial infarct size (for review, see Lochner et al., 2013).

**Figure 2:**
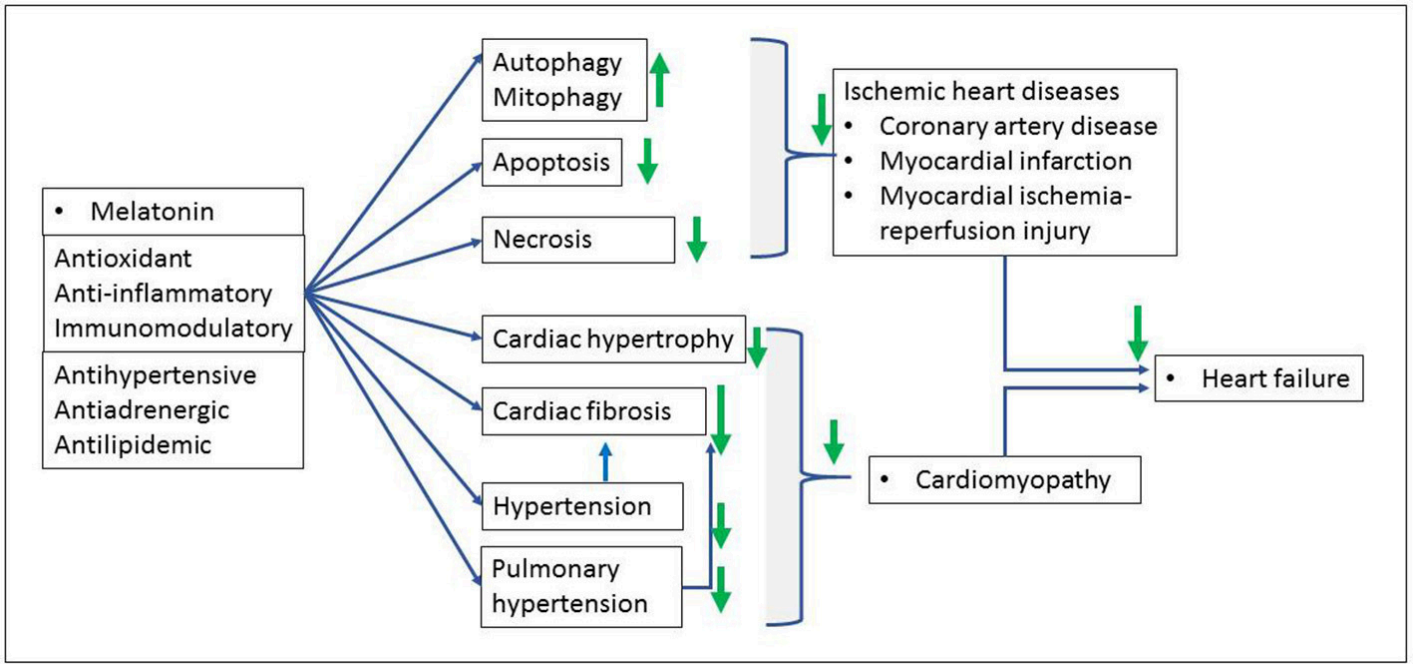
Benefits of melatonin in cardiac pathologies. Melatonin, via its antioxidant, anti-inflammatory and immunomodulatory properties protects against ischemic heart disease as well as subsequent ischemic heart failure characterized by myocardial cell death (necrosis, apoptosis, autophagy) and cardiac dysfunction. Hypertension and pulmonary hypertension induce both cardiac fibrosis and pathological hypertrophy (cardiomyopathy) and subsequent heart failure. Melatonin reverses these effects as indicated with green arrows (↓: increase, ↑: reduce).

The mechanisms of cardioprotection induced with melatonin against myocardial infarction or I/R injury are complex and not well understood. As presented in the Figure 2, melatonin may directly and indirectly affect cardiac pathologies via multiple mechanisms including amongst others, antihypertensive, antilipidemic, antiadrenergic, and immunomodulatory activities. It also reduces oxidative stress, apoptosis, necrosis, mitochondrial permeability transition pore opening, lipid peroxidation and inflammation (for more details, see Yang et al., 2014). Recent findings in the mechanisms of melatonin-induced cardioprotection suggest the involvement of the intracellular survival signaling pathways including, mainly, the activation of the survivor activating factor enhancement (SAFE), the reperfusion injury salvage kinase risk (RISK), Notch1 and sirtuins (SIRT1 and SIRT 3) signaling pathways (Lochner et al., 2013; Nduhirabandi et al., 2014, 2016; Yu et al., 2017b; Zhai et al., 2017a) as well as the crucial role of the mitochondria in cell death and survival (necrosis, apoptosis, autophagy, mitophagy) (Petrosillo et al., 2009; Pei et al., 2016; Hu et al., 2017). The description of these cardioprotective mechanisms is beyond the focus of the present paper (for details, see Lochner et al., 2013; Yang et al., 2014; Sun et al., 2016).

Melatonin supplementation is also beneficial for the treatment of pathological cardiac remodeling and heart failure (Chua et al., 2016; Hu et al., 2017; Zhai et al., 2017b). For example, in a mouse model of myocardial infarction, melatonin significantly reduces adverse left ventricle remodeling and post-myocardial infarction remodeling and dysfunction by increasing autophagy, reducing apoptosis, and reversing mitochondrial dysfunction (Hu et al., 2017). As underlying mechanisms, these effects are associated with a significant activation of adenosine monophosphate-activated protein kinase (AMPK) and an inhibition of macrophage-stimulating 1 (MST) phosphorylation while increasing the expression of SIRT1 and SIRT3, peroxisome proliferator-activated receptor gamma (PPARγ) co-activator 1-protein (PGC-1α), the translocase of the outer membrane 70 (Tom 70), a receptor for translocases in the outer mitochondrial membrane complex (Hu et al., 2017; Pei et al., 2017; Yu et al., 2017b). Furthermore, in a murine model of pathological cardiac hypertrophy induced by transverse aortic constriction (TAC), melatonin supplementation for 4–8 weeks reverses the pathological hypertrophy via the reduction of the pulmonary congestion (Zhai et al., 2017b). These effects are associated with an upregulation of the level of α-myosin heavy chain expression, a downregulation of the levels of β-myosin heavy chain and atrial natriuretic peptide expression, an inhibition of oxidative stress (as expressed by the levels of malondialdehyde (MDA) and superoxide dismutase (SOD) activity), and the activation of PGC-1β as well as the reduction of the cardiac fibrosis (Zhai et al., 2017b). Similar protective effects of melatonin with its antifibrotic properties are also reported in a rat model of cardiorenal syndrome (Chua et al., 2016) and in other animal models of heart failure such as myocardial damage induced by chronic intermittent hypoxia (Yeung et al., 2015), isoproterenol (Patel et al., 2010) or epinephrine (Vazan and Ravingerova, 2015), chemotherapy drugs (Liu et al., 2002), sepsis-induced myocardial injury (An et al., 2016), and diabetic cardiomyopathy (Zhang et al., 2017).

In view of the above benefits in animal studies, melatonin supplementation is highly regarded as an effective therapy in cardiac diseases (Yang et al., 2014; Opie and Lecour, 2016; Sun et al., 2016). Unfortunately, very few clinical trials have investigated the effects of exogenous melatonin in cardiac diseases (Dominguez-Rodriguez et al., 2007; Gogenur et al., 2014; Ekelof et al., 2016) (Table 2). In patients undergoing surgery for abdominal aortic aneurisms, an infusion of 50 mg melatonin over a 2 h period followed by oral administration of 10 mg melatonin for the first three nights after surgery, protects the heart against reperfusion injury by reducing cardiac morbidity as well as the occurrence of myocardial ischemia after abdominal aortic aneurism repair (Gogenur et al., 2014). In patients undergoing coronary artery bypass grafting surgery, melatonin supplementation (10 or 20 mg capsule once a day) increases significantly the ejection fraction and the outcomes together with a remarkable decrease in pro-inflammatory and apoptotic markers, supporting its promising benefits in myocardial I/R injury (Dwaich et al., 2016).

The therapeutic potential of melatonin in cardiac diseases has recently received controversial comments with the publication of the study of melatonin as an adjunct in patients with acute myocardial infarction undergoing primary angioplasty (MARIA trial) (Dominguez-Rodriguez et al., 2007, 2017a,b; Hausenloy et al., 2017). Despite the acceptable safety and tolerability, the MARIA trial reports a lack of cardioprotection in patients treated with formulation of melatonin in polyethylene glycol solution (51.7 μmoles given 60 min before intervention and a bolus of 8.6 μmoles 60 min after intervention) (Dominguez-Rodriguez et al., 2017b). A lack of beneficial effects of melatonin is also reported in other clinical study (Ekeloef et al., 2017). Interestingly, preclinical testing showed that melatonin fails to protect the heart in a closed-chest porcine model of acute myocardial infarction (Halladin et al., 2014; Ekelof et al., 2016) and in a rabbit model of myocardial I/R injury (Dave et al., 1998). It is possible that the intracoronary and intravenous administrations, as opposed to the preferred oral administration, may contribute to the neutral outcomes as suggested by other studies (Dwaich et al., 2016). In addition, ischemic duration and other methodological issues (for review, see Heusch, 2017) may also play a role in the outcomes of above clinical trials, suggesting a re-evaluation of the therapeutic effects of melatonin in a well-planed study.

In the light of the very low physiological concentrations of melatonin, it remains unknown whether consumption of melatonin-rich foods, which increases circulating melatonin concentration into physiological range, may be more effective for cardioprotection.

## Overview of cardiovascular benefits of melatonin-rich food

**Figure 3:**
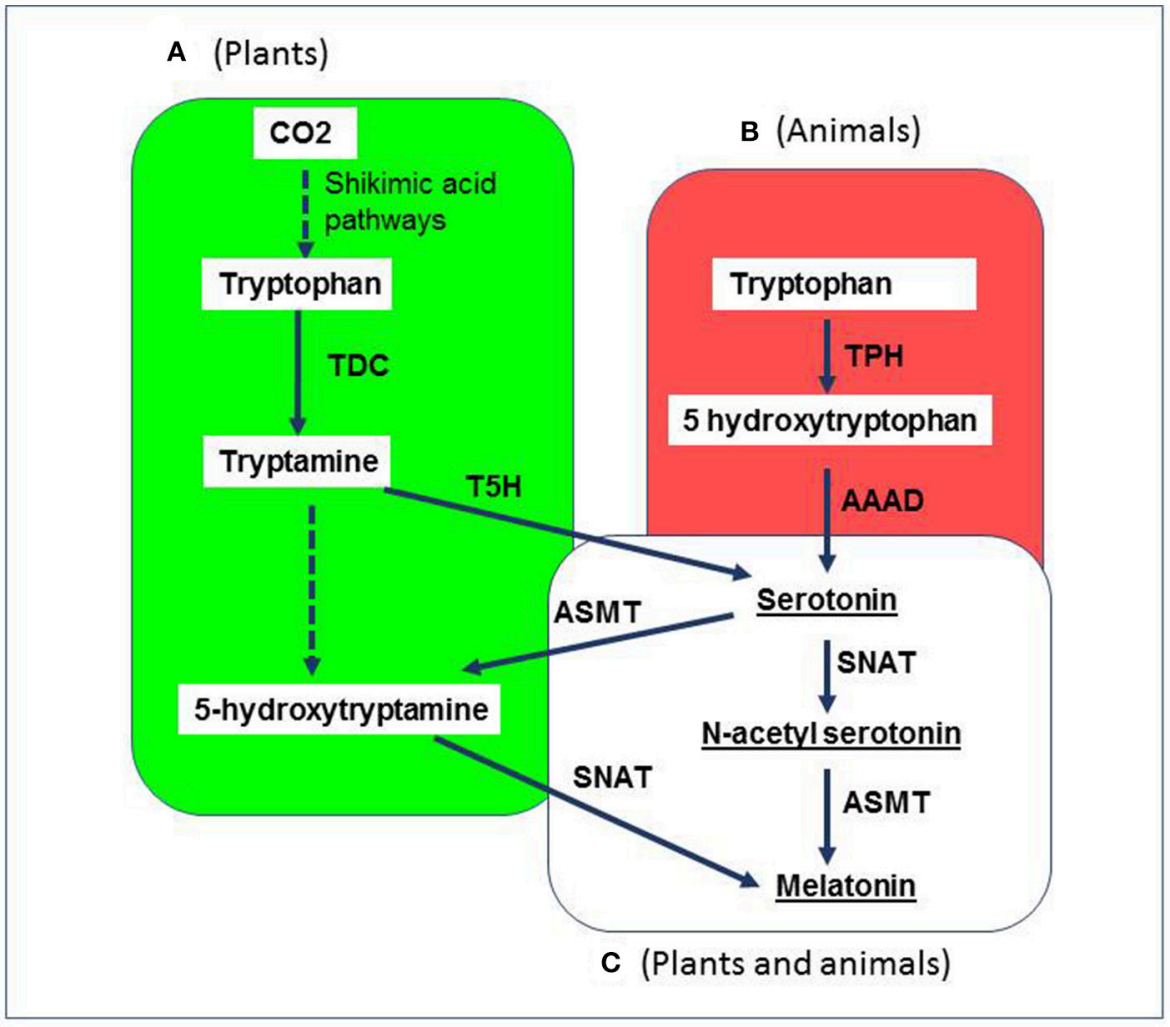
Simplified representation of the biosynthetic pathways of melatonin in plants and animals. Tryptophan is the common precursor of melatonin in all species. **(A)** In plants (green), melatonin is synthesized under two pathways: (1) tryptophan-tryptamine-serotonin-N-acetyl serotonin-melatonin pathway (under normal growth conditions); (2) tryptophan-tryptamine-serotonin-5-methoxytryptamine-melatonin pathway (upon senescence, when plants produce large amounts of serotonin); **(B)** In animals (red); tryptophan is converted in serotonin via hydroxytryptophan; **(C)** In both plants and animals (white), melatonin production from serotonin is the same two-step process and includes the conversion of serotonin to N-acetylserotonin by the rate-limiting enzyme AA-NAT (arylalkylamine N-acetyltransferase) also called as serotonin N-acetyltransferase followed by the conversion of N-acetylserotonin to melatonin by acetylserotonin O-methyltransferase. CO2, carbon dioxide; TPH, tryptophan hydroxylase; AAAD, aromatic amino acid decarboxylase; SNAT, serotonin N-acetyltransferase; ASMT, N-acetylserotonin O-methyltransferase; TDC, tryptophan decarboxylase; T5H, tryptamine 5-hydroxylase.

The presence of melatonin in edible plants and other types of food is well established (Tan et al., 2012, 2014). Melatonin-rich foods include various food components from both animal and plant origins such as chicken, lamb, pork, cow milk, strawberries, tomatoes, olives, grapes, wines, cereals and cherries (for review, see Iriti et al., 2010; Tan et al., 2014) (see Table 3). Interestingly, melatonin concentrations are significantly higher in plants than in animals (Byeon et al., 2014). This is most likely due to differences between the biosynthetic pathways of melatonin in plants and animals (see Figure 3). Plants synthesize tryptophan themselves via the shikimic acid pathway, which increases their melatonin synthetic capacity (Byeon et al., 2014). Animals produce melatonin from tryptophan (essential amino acid from the food) (Byeon et al., 2014). For more details on the biosynthesis of melatonin as well as the dietary source of melatonin, see reviews (Park et al., 2013; Byeon et al., 2014; Tan et al., 2016).

**Table 3:** Some examples of melatonin content in different plants and foods.

**Plant or food**	**Melatonin content**	**References**
Tomato	3–114 ng/g	Stürtz et al., 2011
Walnuts	3–4 ng/g	Reiter et al., 2005
Cereals (rice, barley)	300–1,000 pg/g	Hattori et al., 1995
Strawberry	1–11 ng/g	Iriti et al., 2010
Olive oil	53–119 pg/ml	de la Puerta et al., 2007
Wine	50–230 pg/ml	Murch et al., 2010
Beer	52–170 pg/ml	Maldonado et al., 2009
Cow's milk (unprocessed)	3–25 pg/ml	Májovský et al., 2017
Night-time milk	10–40 ng/ml	Tan et al., 2014
Whole yellow corn	0.28–1.3 ng/g	Tan et al., 2014
Whole chicken meat and skin	0.23–2.3 ng/g	Tan et al., 2014
Chicken heart and liver blend	1.0–1.2 ng/g	Tan et al., 2014

It is likely that the consumption of melatonin-rich food influences endogenous melatonin production (for review, see Peuhkuri et al., 2012). For example, in chickens, circulating melatonin levels increase more than 3.5-folds 1.5 h after the intake of chick food composed of corn, milo, beans, and rice (3 ng/g melatonin) (Hattori et al., 1995). A similar observation is reported in other studies after the consumption of melatonin-rich foods such as walnuts, olive oil, wine, fruits and legumes as well as germinated kidney beans (Peuhkuri et al., 2012; Johns et al., 2013; Sae-Teaw et al., 2013; Tan et al., 2014; Aguilera et al., 2016). However, it remains unclear whether the acute variation of circulating melatonin levels induced by the consumption of melatonin-rich foods correlates with their influence on the cardiovascular system (Bazzano et al., 2001; Al Abdrabalnabi et al., 2017; Aune et al., 2017; Micha et al., 2017). The evidence for cardiovascular benefits of some melatonin containing foods including, walnuts, tropical fruits and grape products are reviewed below.

### Walnuts consumption

Long and short-term observational and intervention studies show that regular consumption of walnuts reduces the risk of CVDs (Kris-Etherton, 2014; Al Abdrabalnabi et al., 2017). In an elderly population, a daily consumption of walnuts improves blood lipids and BP after a one-year trial (Al Abdrabalnabi et al., 2017). In rats fed walnuts an increase in the blood concentration of melatonin and antioxidant capacity is observed (Reiter et al., 2005) However, the correlation between the variation of melatonin levels and the cardiovascular benefits of walnuts remains to be established.

### Tropical fruits consumption

Fruits consumption is associated with reduced CVDs (Aune et al., 2017; Micha et al., 2017). Melatonin is present in tropical fruits, namely banana, pineapple, orange, papaya, mango (Johns et al., 2013). Apart from papaya and mango, the consumption of these fruits increases the circulating melatonin as measured by 6-sulphatoxymelatonin in healthy volunteers (Johns et al., 2013). Interestingly, the rise in serum melatonin levels is positively associated with the antioxidant capacity but not the melatonin content in fruits (Johns et al., 2013; Sae-Teaw et al., 2013). However, it remains unknown whether increasing circulating melatonin levels with fruits consumption is beneficial for patients with reduced levels of endogenous melatonin or CVDs and other illnesses involving oxidative damage.

### Grapes and wine consumption

Chronic and moderate consumption of red wine is associated with a reduced risk of CVDs and other diseases such as diabetes and neurological diseases (Opie and Lecour, 2007). Melatonin is present in grapes and wines; and consumption of grape products may affect endogenous melatonin levels (Iriti, 2009; Murch et al., 2010; Meng et al., 2017a). In young, middle-aged and elderly individuals, intake of 200 mL of grape juice twice a day significantly increases urinary 6-sulfatoxymelatonin and total antioxidant capacity (Gonzalez-Flores et al., 2012). However, whether grape juice-induced elevated circulating melatonin is associated with improved disease conditions is still unknown.

Interestingly, despite the presence of melatonin in grape, melatonin found in wine is mainly synthesized by yeast during alcoholic fermentation (Rodriguez-Naranjo et al., 2011). The concentrations of melatonin in human serum significantly increase after drinking alcoholic beer (Maldonado et al., 2009). Conversely, other studies report a decrease in endogenous melatonin production following alcohol consumption (Peuhkuri et al., 2012) thereby making the influence of alcohol found in wine on melatonin levels inconsistent. Nevertheless, as described below, melatonin present in red wine significantly induces cardioprotective effects (Lamont et al., 2015).

## Role of dietary melatonin in CVDs

Dietary melatonin refers commonly to melatonin content that is present in diet or melatonin-rich food (Iriti and Varoni, 2015; Meng et al., 2017b). Very few studies have investigated the role of dietary melatonin in CVD. These are limited to the exploration of the cardioprotective effect of melatonin in red wine against myocardial I/R injury (Lamont et al., 2011, 2012, 2015; Nduhirabandi et al., 2016) and pulmonary hypertension (Maarman et al., 2015).

### Dietary melatonin and myocardial I/R injury

Using both *in vivo* and *in vitro* models of myocardial I/R, studies from our laboratory demonstrate that the presence of melatonin in red wine may contributes to the protective effect of red wine against lethal I/R damage (Lamont et al., 2011, 2015). This is supported by the findings that: (1) lowering the alcohol content of red wine does not alter its cardioprotective properties (Lamont et al., 2012); and (2) melatonin, given acutely and directly to the isolated heart at the concentration found in wine (75 ng/L) protects the isolated mouse and rat hearts against I/R injury by reducing myocardial infarct size and improving functional recovery (Lamont et al., 2011). (3) The cardioprotective effect of the consumption chronic and moderate red wine against I/R damage is partially abolished in the presence of prazosin, an inhibitor of melatonin receptor type 3 (M3) (Lamont et al., 2015). Further findings from our laboratory show that drinking water supplemented daily with a moderate amount of red wine or melatonin given at the concentration found in the red wine (75 pg/mL) for 14 days protects the rat and mouse hearts subjected to *in vivo* or *ex vivo* I/R by reducing their infarct size (Lamont et al., 2015).

The mechanism of cardioprotection induced with dietary melatonin is complex and still under investigation. As indicated above, melatonin exhibits its physiological functions through its antioxidant, anti-inflammatory, immune-modulatory and vasomotor activities (Pandi-Perumal et al., 2006). Acute (Lamont et al., 2011; Nduhirabandi et al., 2016) and chronic (Lamont et al., 2015) physiological concentrations of melatonin at the concentration found in red wine protect the heart against I/R injury via its membrane receptors related cell survival signaling (Lamont et al., 2015; Nduhirabandi et al., 2016). This is associated with an increased level of activation of myocardial signal transducer and activator of transcription 3 (STAT3) prior to ischemic insult by 79% (Lamont et al., 2011). The importance of STAT3 activation is that it is associated with the activation of SAFE pathway, a well-known powerful cardioprotective signaling pathway (Lamont et al., 2015; Nduhirabandi et al., 2016). Interestingly, luzindole, a specific inhibitor of membrane melatonin receptors 1 and 2 (MT1 and MT 2) (2.3 mg/kg/day, intraperitoneally) does not affect wine treatment, while prazosin, an inhibitor of M3 receptor (2.5 mg/kg/day, intraperitoneally) abolishes wine-induced cardioprotection, therefore suggesting the crucial role of both melatonin and M3 receptor in the cardioprotective effect of red wine (Lamont et al., 2015).

However, how moderate red wine consumption or dietary melatonin affects the SAFE pathway is still unclear. Our recent data suggest that melatonin confers cardioprotection via toll-like receptor 4 (TLR4) which, in turn, activates tumor necrosis factor-α (TNFα)/STAT3 pathway (Nduhirabandi et al., 2016). In view of the pro-inflammatory effects of TLR4 stimulation (Yang et al., 2000), this finding is surprising considering the well-known anti-inflammatory activities of melatonin with its TLR4 suppressing activities (Hu et al., 2013; Mauriz et al., 2013); but it is consistent with the current view of melatonin as an immune system buffer acting as a stimulant under basal or immunosuppressive conditions or as an anti-inflammatory compound in the presence of exacerbated immune responses (for review, see Carrillo-Vico et al., 2013).

### Dietary melatonin and pulmonary hypertension

Pulmonary hypertension is associated with an increased oxidative stress and leads to right ventricle (RV) hypertrophy and cardiac fibrosis with the hallmarks of the heart failure (Maarman et al., 2015). A single subcutaneous injection of monocrotaline (80 mg/kg) induces pulmonary hypertension with RV hypertrophy and dysfunction, increase in interstitial fibrosis and plasma oxidative stress (Maarman et al., 2015). In this model, a chronic dietary melatonin treatment (75 ng/L) reduces RV hypertrophy, improves RV function, reduces plasma oxidative stress and reduces cardiac interstitial fibrosis, therefore supporting the beneficial effects of curative and preventive treatments of dietary melatonin in pulmonary hypertension (Maarman et al., 2015). These effects of melatonin are associated with a reduction in oxidative stress and an increase in enzymatic and non-enzymatic antioxidant capacity (Maarman et al., 2015; Torres et al., 2015).

## Current challenges, limitations and new perspectives for the use of melatonin in CVDs

**Figure 4:**
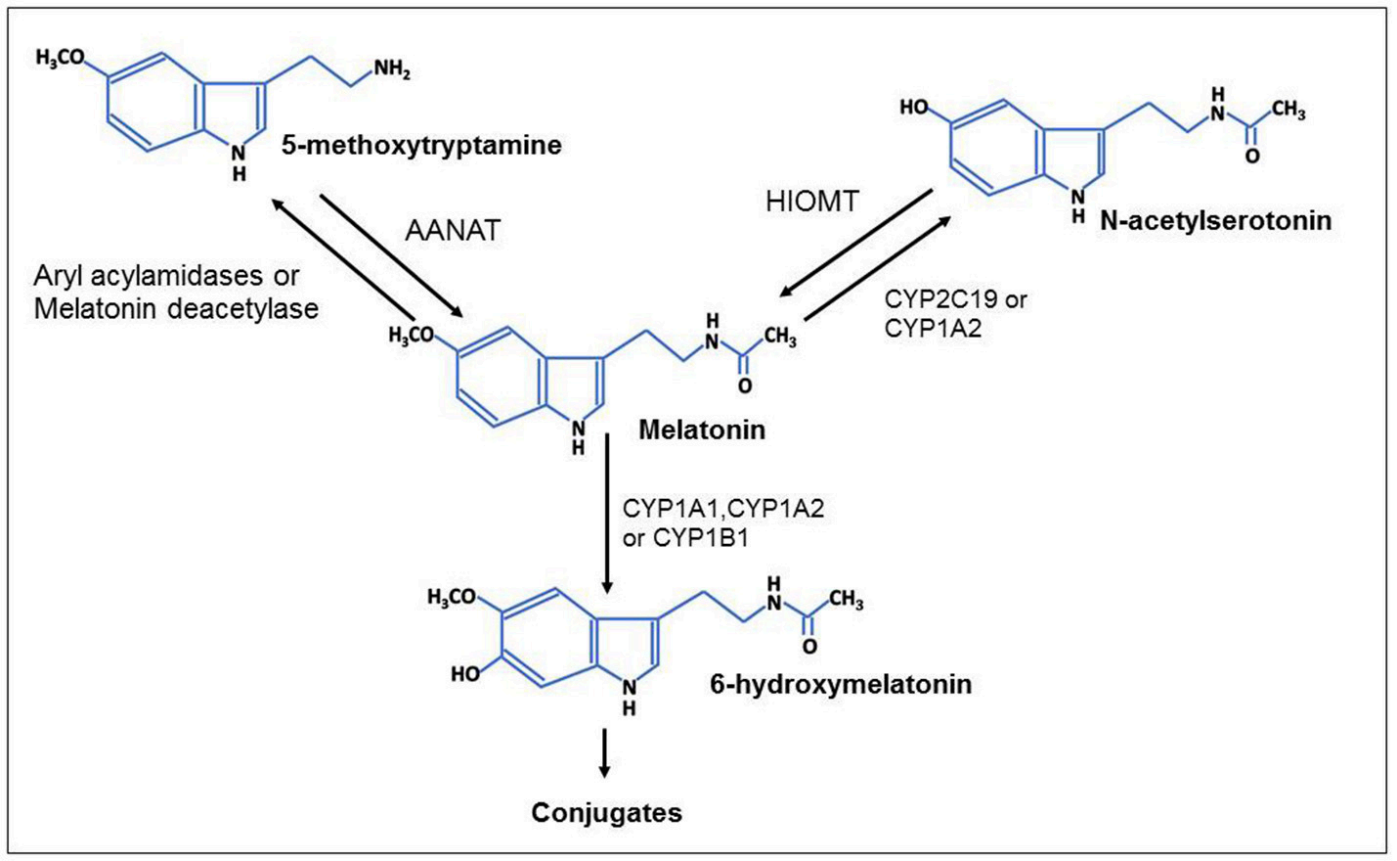
Metabolism of melatonin: enzymatic pathways. Melatonin given orally is principally metabolized in the liver by cytochrome P-450 isoforms (CYP1A1, CYP1A2, and CYP1B1) in 6-hydroxylation to yield 6-hydroxymelatonin which is thereafter conjugated with sulfates to 6-sulfatoxymelatonin and eliminated in urine. Melatonin may also be transformed by deacetylation or by CYP2C19 and CYP1A2 mediated O-demethylation in 5-methoxytryptamine or N-acetylserotonin, respectively. N-acetylserotonin and 5-methoxytryptamine can be converted in melatonin by hydroxyindole-O-methyltransferase (HIOMT) and arylalkylamine N-acetyltransferase (AANAT).

The dietary supplementation of melatonin appears as an effective option to compensate the physiological decline in the production of pineal gland melatonin with ageing; however, the role of dietary melatonin in health and disease is still not well established (Kennaway, 2017; Meng et al., 2017b). The strong limitation for the studies of dietary melatonin is the lack of standardized methods to determine melatonin concentration in foods with adequate sample treatment to obtain accurate results, thus making some of its proposed benefits hard to swallow (Kennaway, 2017). In addition, most of the studies on dietary melatonin measure melatonin content in food, but very few evaluate the bioavailability of melatonin after melatonin-rich food consumption. It is well established that the bioavailability of melatonin after oral and intravenous melatonin administration in human is very low (approximately 15%) with a maximal half-life of 10–60 min (Harpsoe et al., 2015). Although other factors (such as age, disease conditions, specific drugs, cigarette smoking and caffeine intake) may also influence the bioavailability of melatonin which is mostly metabolized by the enzymatic catabolism in the liver (Ma et al., 2005). As shown in the Figure 4, melatonin is principally metabolized in the liver by cytochrome P-450 isoforms (CYP1A1, CYP1A2, and CYP1B1) in 6-hydroxylation to yield 6-hydroxymelatonin which is thereafter conjugated with sulfates to 6-sulfatoxymelatonin and eliminated in urine (Ma et al., 2005; Pandi-Perumal et al., 2006). Melatonin may also be transformed to a less extend by CYP2C19 and CYP1A2 mediated O-demethylation in N-acetylserotonin (N-acetyl-5-hydroxytryptamine) or by deacetylation to 5-methoxytryptamine which can be reconverted to melatonin (Ma et al., 2005).

Furthermore, how melatonin-rich food contributes to the overall endogenous melatonin production is complex and still unclear. Besides the melatonin content in the food, food intake may also influence endogenous melatonin concentration, suggesting that the overall beneficial effect could be a result of a combined action of more components found in these food stuffs. Indeed, an increase in blood melatonin levels may be a result of other components found in food that may stimulate endogenous melatonin production such as in the case of ingestion of tryptophan (Huether et al., 1992; Bravo et al., 2013) or serotonin (Esteban et al., 2004). These possible effects make more challenging the delimitation of the contribution of the dietary melatonin to the overall cardiovascular benefits of melatonin-rich foods. Moreover, to avoid bias due to drug interaction, the drugs that are metabolized by cytochrome P-450 such as fluvoxamine, caffeine, and oral contraceptives need to be considered (Harpsoe et al., 2015). Since these drugs compete with melatonin for the same enzyme, they may increase the plasma levels of melatonin after exogenous melatonin administration (Harpsoe et al., 2015).

Due to the very low bioavailability of melatonin after oral or intravenous administration, new developments to optimize the intake of melatonin as supplement or food consider various alternative administrative routes, namely: intranasal, transdermal, subcutaneous, and oral transmucosal (such as sublingual and trans buccal) administration or other forms of preparations (spray, elastic liposomes, gels, pastes) (for review, see Zetner et al., 2016). These alternative routes are very important because they: (1) bypass the liver metabolism, (2) are painless, and (3) provide possible sustained release with a subsequent increase in the bioavailability of melatonin (Zetner et al., 2016).

Although melatonin supplementation is safe in patients with myocardial infarction (Dominguez-Rodriguez et al., 2017b) and nocturnal hypertension (Grossman et al., 2011; Simko et al., 2016), melatonin is contra-indicated for patients with high normal BP to avoid the danger of the diurnal arterial hypertension (Rechcinski et al., 2010). In addition, in children with age of 1-year, increased serum melatonin levels may be associated with severe heart failure (Wu et al., 2017), raising the alarming issue of a high dose of melatonin supplementation in children at this age. While this association is surprising in view of low circulating melatonin levels in adult patients with heart failure, it may be explained by either compensatory mechanisms (of heart failure) or potential detrimental effects of high dose of melatonin in infants (Wu et al., 2017).

Thus far, there is no evidence for the dose-dependent effect of melatonin in the context of cardiovascular diseases. Current data show that both high and low doses of short-term melatonin treatment confer cardiovascular protection in experimental animal models. According to recent clinical trials, although the overall melatonin treatment is safe, it may cause mild adverse effects from transient sedation, daytime sleepiness, mild headache to worsening of dyspnea, and combining melatonin with any drug associated with these effects is contra-indicated (for review, see Andersen et al., 2016). However, further investigation is needed to determine the optimal dose of melatonin and the effects of long-term of melatonin supplementation in humans.

Recent data from clinical studies using melatonin in cardiac diseases show more inconsistencies regarding its cardioprotective effects (Andersen et al., 2016; Dominguez-Rodriguez et al., 2017b; Ekeloef et al., 2017). Apart from dosage issues and mode of administration, previous failures could be partially explained by the use of young and healthy animals with eventual lack of various cardiovascular risk factors, comorbidities and comedications which are characteristics of patients suffering an acute myocardial infarction or undergoing cardiovascular surgery (Heusch, 2017). Considering the current disappointment, further well-planned preclinical and clinical studies are needed to better delineate the cardiovascular benefits of melatonin.

## Conclusion

In summary, preclinical studies clearly suggest the cardiovascular health benefit of both endogenous and supplementary melatonin. Melatonin is an important safe molecule with a wide range of physiological functions in animals and humans with a strong therapeutic potential in CVDs. Despite the current difficulties to translate the basic research findings into a clinical setting, cardiovascular protective action of melatonin supplementation is promising but a better understanding of this action is needed. Most importantly, consumption of melatonin-rich foods such as grape juice, wine, cereals, tropical fruits and walnuts increases circulating melatonin levels and antioxidant capacity. Preclinical studies suggest that melatonin, given at dietary levels, confers cardioprotection. However, a better understanding of the mechanisms involved in this effect are required before it can be considered as an adjuvant for effective preventive and curative therapy in CVDs.

## Author contributions

ZJ: wrote the first draft of the manuscript; SL: conception, critical comments on the different drafts of the manuscript (content review); FN: conception, literature choice, figures and tables, revised the final draft manuscript.

### Conflict of interest statement

The authors declare that the research was conducted in the absence of any commercial or financial relationships that could be construed as a potential conflict of interest.
